# Cascade *Fumarate Hydratase* mutation screening allows early detection of kidney tumour: a case report

**DOI:** 10.1186/s12881-017-0436-1

**Published:** 2017-07-26

**Authors:** Melanie M. Y. Chan, Angela Barnicoat, Faiz Mumtaz, Michael Aitchison, Lucy Side, Helen Brittain, Alan W. H. Bates, Daniel P. Gale

**Affiliations:** 1 Centre for Nephrology, University College London, Royal Free Hospital, London, UK; 2 Northeast Thames Regional Genetics Service, Great Ormond Street Hospital for Children, London, UK; 3 Department of Urology, Royal Free Hospital, London, UK; 4 Department of Histopathology, Royal Free Hospital, London, UK

**Keywords:** Fumarate hydratase deficiency, Hereditary leiomyomatosis and renal cell cancer (HLRCC), Hereditary cancer, Case report

## Abstract

**Background:**

Fumarate hydratase (FH) deficiency is a rare autosomal recessive disorder which results in a major defect in cellular metabolism. It presents in infancy with progressive encephalopathy, hypotonia, seizures and failure to thrive and is often fatal in childhood. It is caused by mutations in the *FH* gene (1q42.1) that result in deficiency of the citric acid cycle enzyme fumarate hydratase, resulting in accumulation of fumaric acid. Heterozygous germline mutations in the *FH* gene predispose to an aggressive autosomal dominant inherited early-onset kidney cancer syndrome: hereditary leiomyomatosis and renal cell cancer (HLRCC).

**Case presentation:**

Cascade *FH* mutation screening enabled the early diagnosis of a renal tumour in an asymptomatic parent of a child with fumarate hydratase deficiency, resulting in timely and possibly life-saving treatment.

**Conclusion:**

While the theoretical risk of kidney cancer in parents of children with recessive fumarate hydratase deficiency is well recognized, to our knowledge this is the first report of a kidney tumour being detected in a parent by screening performed for this indication. This underscores the importance of offering lifelong kidney surveillance to such parents and other heterozygous relatives of children born with fumarate hydratase deficiency.

## Background

Inherited cancer syndromes are an important cause of disease, accounting for 5-10% of cancers in the general population [1]. These disorders are usually inherited as dominant traits and therefore afflict multiple generations of the same family, either in childhood or adulthood. Early diagnosis can have major survival benefits and active surveillance for cancer from a young age allows tumors to be safely removed before they have metastasized. A detailed family history is therefore a crucial part of the assessment of all patients with cancer.

Identification of the mutated genes responsible for inherited cancer syndromes is important because bi-allelic somatic mutations in the same genes are frequently found in sporadic cancers; a major cause of death in the general population. Defining these genes has provided some key insights into the cellular mechanisms responsible for cancer.

Kidney cancer kills approximately 4250 people each year in the UK (~3% cancer deaths) [2]. It frequently presents late, often only detected incidentally during radiological imaging of the abdomen, and if it has metastasized at time of diagnosis, may prove fatal. Early detection of renal lesions in at-risk individuals can therefore be life-saving.

Here, we present a case of a kidney tumour associated with hereditary leiomyomatosis and renal cell cancer (HLRCC) that was diagnosed by cascade screening of the healthy parents of an infant with autosomal recessive fumarate hydratase deficiency.

## Case presentation

The index case is the first child of unrelated healthy Caucasian parents. Antenatal ultrasound identified the child had dilated cerebral ventricles and she was induced at term for this reason. She had an uncomplicated delivery and was of normal birth weight. There was initial concern about jaundice but no specific treatment was required.

She presented at 3 months of age with seizures and hypotonia although her parents had noted some visual inattentiveness and developmental delay prior to presentation. Seizure control was challenging with short periods of good control being achieved with multiple anticonvulsant agents. On examination, she was plagiocephalic, with an alternating divergent strabismus, a wide mouth with tented upper lip and some facial coarsening. An MRI scan showed a delay in myelination and evidence of poor white matter bulk.

Urinary organic acid analysis by gas-chromatography mass spectrometry showed raised fumarate (4790 μmol/mmol creatinine) with mildly raised succinate and 2-oxoglutarate levels. Functional enzyme analysis in fibroblasts showed fumarate hydratase activity of 20% control level, and genetic analysis of the *FH* gene showed compound heterozygosity for mutations in c.844G>C p.Gly282Arg (present in the father) and c.1127A>C p.Gln376Pro (inherited from the mother), confirming a diagnosis of fumarate hydratase deficiency.

Developmental progress was very slow. She was babbling and rolling at 2 years of age but did not achieve independent sitting and her development plateaued from around 2.5 years. She had significant central visual impairment limited to perception of light. There were episodes of status epilepticus and latterly episodes of abnormal posturing and dystonia. Her growth faltered and she failed to thrive, her weight fell below the 0.4th centile and she died at 4 years of age.

**Fig. 1:**
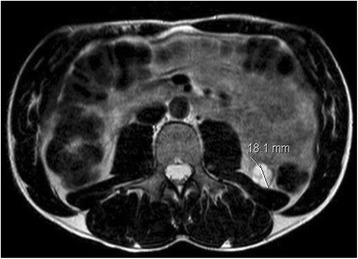
Abdominal MRI showing a 2 × 2 × 1.8 cm exophytic lesion in the lower pole of the left kidney demonstrating septation and enhancement

**Fig. 2:**
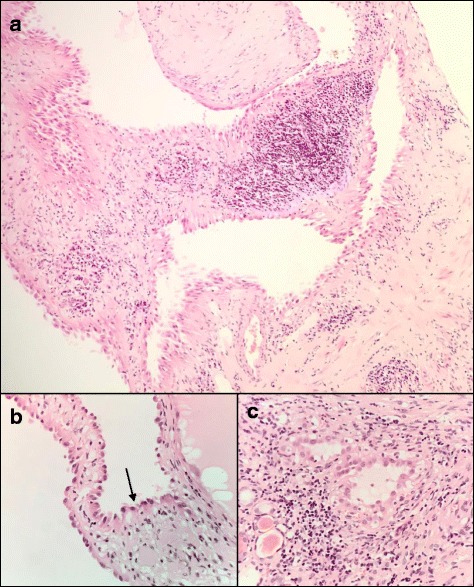
Histology shows a multicystic tumour with cysts lined by hobnailed cells (*arrow*) with a low mitotic index and prominent nucleoli (**a**, low power; **b**, high power). Within the stroma are tubular elements with similar nuclear features (**c**)

The parents were offered screening by renal ultrasonography in view of their known heterozygous mutations in the *FH* gene. An ultrasound of the 30-year-old asymptomatic father showed a cystic lesion at the lower pole of the left kidney. MRI scan confirmed a 2 cm × 2 cm × 1.8 cm cystic exophytic lesion (Fig. 1). There was no evidence of metastatic spread and he underwent a robot-assisted laparoscopic partial left nephrectomy from which he made an uneventful recovery. Histological examination of the renal lesion demonstrated a low grade tubulocystic carcinoma, with cysts lined by hob-nailing cells containing eosinophilic cytoplasm and rounded nuclei with conspicuous nucleoli, which had been completely resected (Fig. 2). Immunohistochemistry showed positivity for CD10, vimentin and CK19 and was negative for CK7, racemase, and RCC. FH and 2-Succinocysteine (2SC) immunohistochemistry was not assessed in line with current routine clinical practice.

## Discussion and conclusions

Approximately 3-5% of all kidney cancers are inherited and at present there are ten syndromes and 12 genes that have been found to be associated with an increased risk of kidney cancer [3]. Heterozygous mutations in genes including *VHL*, *Fumarate hydratase* (*FH*) and *Succinate dehydrogenase* subunits (*SDHB*, *SDHC* and *SDHD*) are associated with different types of renal cancers and somatic loss of the second allele can often be demonstrated in the associated renal lesions. Many of the genetic defects associated with inherited kidney cancer syndromes perturb cellular metabolism by disrupting the citric acid cycle and the renal lesions associated with these different molecular defects have different histological and clinical characteristics. Here we present a case of an *FH*-associated kidney tumour identified by screening an asymptomatic parent of a child with autosomal recessive fumarate hydratase deficiency caused by compound heterozygous mutations in the *FH* gene.

Heterozygous loss-of-function *FH* mutation is associated with hereditary leiomyomatosis and renal cell cancer (HLRCC) syndrome [4]. The clinical manifestations include uterine fibroids (present in 75-98% of female mutation carriers) often requiring myomectomy or hysterectomy before the age of 40 years [4–6]; painful cutaneous leiomyomata (present in 76-100% of mutation carriers, with mean age of onset of 25 years) [5, 7, 8]; and renal cell carcinomas (RCCs) which occur with variable penetrance in approximately 14-18% of affected individuals [7, 9]. Following the recognized association with renal cancer, the term HLRCC has superseded the earlier terms Reed’s Disease [10] and Multiple Cutaneous and Uterine Leiomyomata syndrome (MCUL) [4].

HLRCC-associated RCCs are a group of histologically heterogeneous tumors that can be described as papillary, solid, tubulocystic, cribiform or cystic in nature and have recently been added as a separate entity to the 2016 WHO Classification [11]. Cells share a characteristic appearance; a large nucleus and prominent inclusion-like eosinophilic nucleoli surrounded by a clear halo [12]. Immunohistochemistry demonstrates a lack of FH protein expression and increased 2SC levels in the tumor [13–15]; a finding that should prompt genetic screening of affected individuals. Traditionally ‘type 2 papillary RCCs’ were the renal lesion most commonly associated with HLRCC, however a recent genomic analysis has suggested that ‘type 2 papillary RCC’ is not in fact a single tumor type, but instead consists of sub-groups with different molecular backgrounds [16]. Interestingly, DNA methylation analysis identified a CpG island methylator phenotype (CIMP) which was associated with reduced *FH* mRNA expression; this was seen in 56% of tumors with germline FH mutations and associated with poor survival [16].

It is clinically important to differentiate FH-deficient RCC from other renal cell cancers as the former have a high chance of early invasion and metastasis, even when the lesion diameter is very small (<1 cm). This contrasts with most sporadic renal cell carcinomas and those associated with other inherited conditions (such as VHL disease), in which tumours smaller than 3 cm in diameter tend to be well circumscribed with a very low probability of metastasis. This distinction suggests that aggressive screening (by regular high-resolution imaging) and early resection of even very small suspicious lesions that are identified in patients with HLRCC may be necessary to reduce the risk of fatal metastatic cancer [17]. Management of these lesions requires early nephron-sparing surgery and a Phase II clinical trial (NCI 10-C-0114) for the treatment of advanced or metastatic HLRCC-associated kidney cancer using bevacizumab (targeting VEGFA) and erlotinib (targeting EGFR kinase activity) is currently under way (NCT01130519).

Germline mutations in the *FH* gene are diagnostic for HLRCC and approximately 120 potentially pathogenic mutations have been identified [18]. Genetic testing should be offered to anyone with clinical manifestations of the syndrome or with a family history of HLRCC. The renal tumours seen with HLRCC can present at a young age (10-44 years) and current recommendations suggest considering screening children with a known heterozygous *FH* mutation with abdominal MRI annually from the age of 8 years [19].

The *FH* gene has been suggested as a ‘non-classical’ tumour suppressor gene. It encodes the enzyme fumarate hydratase which catalyses the conversion of fumarate to malate in the citric acid cycle. Deficiency of this enzyme results in excessive fumarate accumulation and it is hypothesised that fumarate may act as an ‘oncometabolite’ in *FH*-deficient kidney cancer [17]. One theory is that the excess fumarate induces an environment of ‘pseudo-hypoxia’ by stabilisation of the transcription factor hypoxia-inducible factor 1-alpha (HIF-1α) and upregulation of its target genes vascular endothelial growth factor (VEGF) and glucose transporter 1 (GLUT1) creating optimal conditions for tumour proliferation. Alternatively, it has been proposed that fumarate may indirectly stabilise the transcription factor nuclear factor erythroid 2-related factor 2 (Nrf2) and result in activation of the Nrf2 antioxidant pathway [20].

While HLRCC is caused by heterozygous *FH* mutations, and transmitted as an autosomal dominant trait, inheritance of two defective *FH* alleles has been described in around 100 patients, and is associated with a significant deficiency of the fumarate hydratase enzyme. This causes a major defect of cellular metabolism which usually manifests in the peri-natal period or in early infancy. Dysmorphology is frequently present and characteristic features include a depressed nasal bridge, widely spaced eyes, frontal bossing, ear anomalies and narrow forehead. Brain atrophy and white matter abnormalities are also described [21]. Clinical features include encephalopathy, poor feeding, lethargy, stupor and epilepsy. Other reports include recurrent vomiting and hepatosplenomegaly [21], neonatal polycythaemia [22] and pancreatitis [23]. Clinical outcomes in these infants are unfortunately poor with progressive neurological and systemic disease leading to death in infancy or early childhood in most cases [24].

While the theoretical risk of kidney cancer in parents of children with recessive fumarate hydratase deficiency is well recognized, to our knowledge this is the first report of identification of a parent with a kidney tumour by screening performed for this indication. This underscores the importance of offering lifelong kidney surveillance to such parents and other heterozygous relatives of children born with fumarate hydratase deficiency.

## Abbreviations

2SC: 2-Succinocysteine
EGFR: Epidermal growth factor receptor
FH: Fumarate hydratase
GLUT1: Glucose transporter 1
HIF-1α: Hypoxia-inducible factor 1-alpha
HLRCC: Hereditary leiomyomatosis and renal cell cancer
MCUL: Multiple cutaneous and uterine leiomyomata syndrome
MRI: Magnetic resonance imaging
Nrf2: Nuclear factor erythroid 2-related factor 2
RCC: Renal cell carcinoma
*SDHB*, *SDHC* and *SDHD*: *Succinate dehydrogenase* subunits
VEGFA: Vascular endothelial growth factor A
VHL: Von-Hippel Lindau
